# Genomic epidemiology describes introduction and outbreaks of antifungal drug-resistant *Candida auris*

**DOI:** 10.1038/s44259-024-00043-6

**Published:** 2024-09-30

**Authors:** Dana Kappel, Hugh Gifford, Amelie Brackin, Alireza Abdolrasouli, David W. Eyre, Katie Jeffery, Silke Schlenz, David M. Aanensen, Colin S. Brown, Andrew Borman, Elizabeth Johnson, Alison Holmes, Darius Armstrong-James, Matthew C. Fisher, Johanna Rhodes

**Affiliations:** 1 MRC Centre for Global Disease Analysis, Imperial College London, London, UK; 2 MRC Centre for Medical Mycology, University of Exeter, Exeter, UK; 3 Department of Medical Microbiology, King’s College Hospital, London, UK; 4 Oxford University Hospitals NHS Foundation Trust, Oxford, UK; 5 Big Data Institute, Nuffield Department of Population Health, University of Oxford, Oxford, UK; 6 School of Immunology and Microbial Sciences, King’s College London, London, UK; 7 Centre for Genomic Pathogen Surveillance, University of Oxford, Oxford, UK; 8 Royal Free London NHS Foundation Trust, London, UK; 9 National Institute for Health Research (NIHR) Health Protection Research Unit in Healthcare Associated Infections and Antimicrobial Resistance, Imperial College London, London, UK; 10 National Mycology Reference Laboratory, UK Health Security Agency, Bristol, UK; 11 Medical Research Council Centre for Medical Mycology (MRC CMM), University of Exeter, Exeter, UK; 12 Department of Infectious Diseases, Imperial College London, London, UK; 13 Department of Medical Microbiology, Radboudumc, Nijmegen, the Netherlands

**Keywords:** Evolutionary genetics, Fungal genomics, Fungal evolution, Eukaryote, Evolutionary ecology

## Abstract

*Candida auris* is a globally emerged fungal pathogen causing nosocomial invasive infections. Here, we use cutting-edge genomic approaches to elucidate the temporal and geographic epidemiology of drug-resistant *C. auris* within the UK. We analysed a representative sample of over 200 isolates from multiple UK hospitals to assess the number and timings of *C. auris* introductions and infer subsequent patterns of inter- and intra-hospital transmission of azole drug-resistant isolates. We identify at least one introduction from Clade I and two from Clade III into the UK, and observe temporal and geographical evidence for multiple transmission events of antifungal drug resistant isolates between hospitals and identified local within-hospital patient-to-patient transmission events. Our study confirms outbreaks of drug-resistant *C. auris* are linked and that transmission amongst patients occurs, explaining local hospital outbreaks, and demonstrating a need for improved epidemiological surveillance of *C. auris* to protect patients and healthcare services.

## Introduction

The ascomycete yeast *Candida auris* was first described in 2008 colonising the ear canal of a Japanese patient^1^. This fungus has now emerged as a global pathogen which has been identified in over 40 countries worldwide^2–4^. Although often misidentified as other *Candida* species (including *C. haemulonii*), *C. auris* infections were very rare prior to 2009^3,5^, with the current global distribution explained by spatial emergence from a small number of foci. Recently, coastal waters were proposed as a potential environmental niche, with *C. auris* isolated from Andaman Islands coastal wetlands^6^ and Colombian estuaries^7^, but spread could have spread into aquatic environments from contaminated wastewater^8^. *C. auris* can easily spread in healthcare settings, to persist for long periods, to colonise human skin, and to survive for weeks on inanimate surfaces and objects. These features of *C. auris* have led to high rates of nosocomial transmission, with outbreaks in hospitals and other healthcare facilities around the world^9–14^. Whilst the intrinsic antifungal resistance of *C. auris* to fluconazole is not unusual amongst yeasts, the propensity to develop resistance to additional azole agents and also other antifungal classes make it unusual among *Candida* species^9,15,16^. And, while *C. auris* mainly colonises skin, the fungus can also cause life-threatening invasive infections^17,18^. *Candida* blood stream infections (candidaemia), are the fourth most common nosocomial bloodstream infection, and are thought to remain as prominent, despite changing epidemiology. For hospitalised patients with comorbidities, mortality rates are estimated between 35% and 45% within the first month of diagnosis with *C. auris* candidaemia, illustrating the serious nature of the infection^19,20^. *C. auris* spread in nosocomial settings has been shown to be through contact with contaminated environmental surfaces, fomites or equipment (such as reusable temperature probes, portable ventilators and pulse oximeters), or by person-to-person contact^9,13–15,19,21,22^. Patient movement between hospitals for rehabilitation or further treatment is thought to contribute to the spread of *C. auris* regionally and between local networks of healthcare facilities^23,24^. In other countries, nationwide transmission has also been identified with genetically similar or highly identical isolates in India and Colombia being found in different hospitals, hundreds to thousands of miles apart^15,21^.

Research has identified the near-simultaneous emergences of four distinct clades of *C. auris*^5^. These clades are named after their inferred geographic region of origin: South Asian (Clade I), East Asian (Clade II), African (Clade III) and South American (Clade IV), although it is problematic to refer to them based on geography^3^. Whilst not currently found as extensively, the fifth and sixth clades have been described^25–28^, and it is not improbable more will be discovered^3^. Whilst there is low within-clade diversity, inter-clade diversity is high, with isolates between clades differing by thousands of single nucleotide polymorphisms (SNPs)^29^. *C. auris* exhibits intrinsic resistance to the antifungal drug fluconazole with clade-associated point mutations in the *ERG11* gene (encoding lanosterol 14α-demethylase) associated with resistance to this drug: Y132F and K143R in Clade I, F126L in Clade III and Y132F and K143R in Clade IV^5,23,30–32^. Clade II isolates, as well as the isolate from Iran (termed Clade V), are generally fluconazole-susceptible, with clade-specific patterns of azole antifungal resistance^5^, and varying susceptibility to amphotericin B, echinocandins and 5-flucytosine^15,31–33^.

With many isolates being multidrug-resistant (MDR) and some being extensively drug-resistant (XDR), *C. auris* is a priority pathogen in the global antimicrobial resistance (AMR) agenda^21,34–36^ and has recently been included as a priority pathogen by the WHO^37–39^. Since 2015, the United Kingdom (UK) has seen several outbreaks in National Health Service (NHS) specialist and tertiary hospitals^9,13,40^. Retrospective analysis of historical isolates at the UK National Mycology Reference Laboratory (MRL) failed to find evidence of *C. auris* in the UK prior to 2013^41^; and whilst multiple introductions of *C. auris* have been identified^42^, to date, the molecular epidemiology of *C. auris* across the UK as a whole remains largely uncharacterised. At a more global scale, analysis of isolates from outbreaks and individual cases has identified countries that have isolates from different clades circulating, suggesting multiple introductions from different origins, followed by local transmission^30^. In this study, we use a genomic framework to evaluate the epidemiology of *C. auris* within the UK in order to determine the number and timing of introductions into the country and to more fully understand how this pathogen transmits and spreads within and between healthcare settings.

## Results

Two hundred and seven *C. auris* isolates consisting of a representative sample of cases collected from patients at nine hospitals between June 2014 and May 2019 in the UK, including the 67 newly sequenced isolates, were aligned to the *C. auris* reference genome, B8441 (Clade I). Out of the 207 isolates, 114 (55%) were from Clade I and 93 (45%) were from Clade III, illustrated by high sequence divergence away from Clade I (Supplementary Fig. 2). Of the sequenced isolates, 187 were isolated from patients, 13 from hospital environments and 7 were of unknown origin. In two hospitals (King’s College Hospital (KCH) and Centre E, isolates from both clades were co-circulating. Clade III isolates were subsequently realigned to the B11221 (Clade III) reference genome. The mean genome coverage was 61× (range 15×–227×) for Clade I and 45× (range 7×–118×) for Clade III. On average, for Clade I, 99.14% and for Clade III, 98.22% of all reads were successfully mapped to their respective reference genomes. After two filtering steps to retain only high-confidence SNPs, on average, for Clade I and Clade III, 94.2% and 92.9% SNPs were kept.

**Fig. 1:**
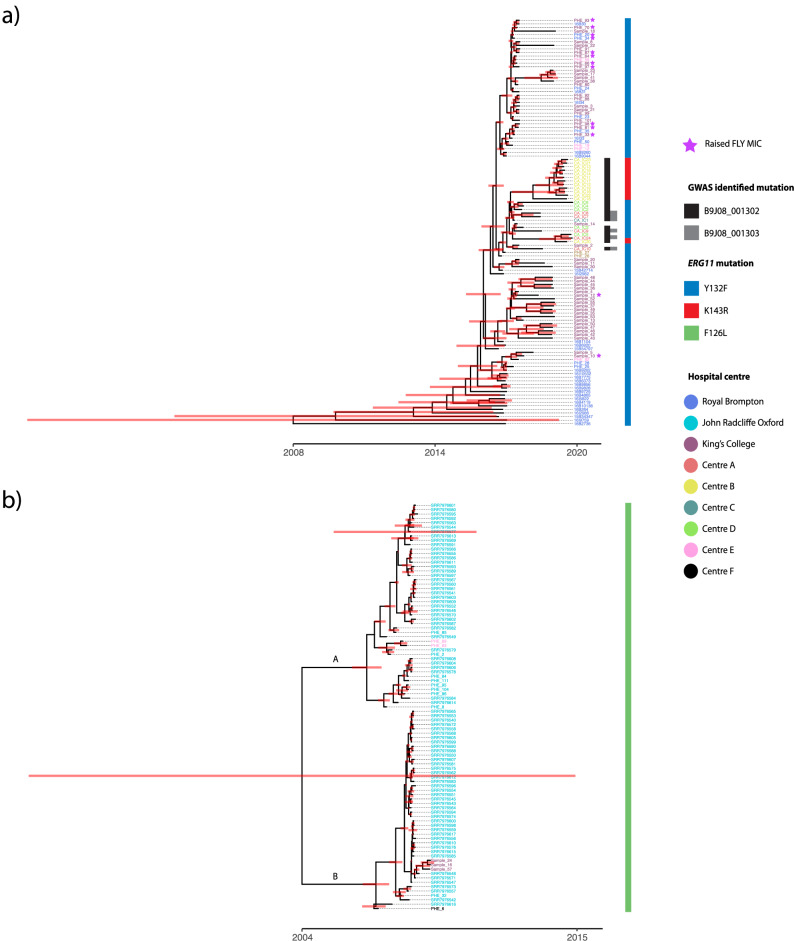
Dating the introduction of *C. auris* into the United Kingdom. *ERG11* mutations are shown for each isolate. **a** Maximum clade credibility phylogeny using the posterior tree distributions of UK Clade I isolates showing a TMRCA as 2008 (CI: 1989–2015). **b** Maximum clade credibility phylogeny using the posterior tree distributions of UK Clade III isolates showing a TMRCA as 2004 (CI: 1972–2015). Subclades A and B are denoted and posterior distributions are shown. Isolates with raised MICs to 5-FC are depicted with a purple star, and ERG11 mutations are shown as blue for Y132F, red for K143R, and green for F126L. Mutations associated with fluconazole resistance, according to previous studies, are also shown, namely Ala224Ala in B9J08_001302 and His283His in B9J08_001303.

Nonsynonymous substitutions within the coding regions of the *ERG11*, *FKS1* and *FUR1* genes were evaluated to search for known mutations conferring resistance to fluconazole, echinocandins and flucytosine antifungal drugs, respectively. Fluconazole resistance-associated mutations in the *ERG11* region were found in all isolates (Fig. 1); 33% (*n* = 69) of isolates have been MIC tested for fluconazole (Supplementary Table 1), and all displayed fluconazole resistance. Of the 207 isolates, 44.9% (*n* = 93) had the F126L *ERG11* substitution, of which all isolates were in Clade III; while 6.3% (*n* = 13) had the K143R *ERG11* substitution, and 48.8% (*n* = 101) had the Y132F *ERG11* substitution and were found in Clade I (Table 1). The point mutations in the *ERG11* gene in the UK are clade-specific, with Clade I exhibiting K143R and Y132F and Clade III exhibiting F126L substitutions. This distinction does not expand globally as Clade IV has the same resistance mutations in *ERG11* as Clade I^5,29,30^. A recent study performed genome-wise association studies (GWAS) on nearly 400 isolates with whole genome sequences and accompanying antifungal susceptibility profiles^43^. Out of the 15 SNPs identified to be associated with fluconazole resistance, we found two present in our data: a synonymous mutation (Ala224Ala) in B9J08_001302 present in 24 isolates, and another synonymous mutation (His283His) in B9J08_001303 present in six isolates (Fig. 1). None of the mutations identified in the study as being associated with raised MICs to other antifungal drugs was seen in our Clade I isolates. All Clade III isolates also contained the Y125A substitution within *ERG11*. This allele has not been found previously in *C. auris*. None of the Clade III isolates contained the mutations previously identified as being associated with antifungal drug resistance *via* GWAS^43^. Analysis of the *FKS1* and *FUR1* genes showed no mutations. However, 13 isolates displayed elevated MICs for flucytosine, which may be indicative of drug resistance, suggesting alternate mechanisms other than *FUR1* being responsible (Fig. 1). These isolates were all Clade I and were only found in King’s College and Royal Brompton hospitals, yet were not confined to a single sub-clade of the Clade I phylogeny (Fig. 1). None of the isolates with completed antifungal susceptibility testing displayed raised MICs to echinocandin antifungal drugs. A summary of the antifungal susceptibility data and known resistance mutations can be found in Supplementary Table 1.

**Table 1:** Summary of Clade I and Clade III isolates included within this study and details on *ERG11* polymorphisms

	Number of isolates (%)	Mating type	Number of hospitals	Mutations in the *ERG11* region (number of isolates)	Source (number of isolates)	Mean genome coverage	Percentage successfully mapped to reference genome	Percentage unfiltered vs. filtered SNPs
Clade I	114 (55.1%)	MTLa	8	Y132F (*n* = 101) K143R (*n* = 13)	Clinical (86) Environmental (6) Unknown (1)	61×	99.14	94.2
Clade III	93 (44.9%)	MTLα	3	F126L (*n* = 93) Y125A (*n* = 93)	Clinical (101) Environmental (7) Unknown (6)	45×	98.22	92.9
UK Total	207 (100%)		9		Clinical (187) Environmental (13) Unknown (7)	54×	98.73	94.2

### Temporal analysis shows at least three introductions of *C. auris* into the United Kingdom

Maximum likelihood (ML) trees inferred using whole-genome SNPs for Clade I and III were used as the input to calculate root-to-tip regressions for each clade (Supplementary Fig. 1). The analysis included all 114 Clade I and 93 Clade III isolates from the UK dataset, with the date of sample collection used for tip dating. We observed a positive correlation between the tip dates and divergence from the root using TempEst for Clade I (*R*^2^ = 0.38, *p*-value 1.93e^−13^), and Clade III (*R*^2^ = 0.53, *p*-value 6.19e^−17^). Phylostems estimated the TMRCA corresponding to the *x*-intercept of the regression, indicating that the MRCA for Clade I and Clade III occurred in 2000 and 2013, respectively, representing the most likely year of entry into the UK if each clade was only introduced on a single occasion.

To refine this estimate, the dataset was subjected to coalescence analysis using BEAST. Using the posterior distributions of the Clade I and III phylogenies, maximum credibility clade (MCC) trees were inferred to analyse introductions into the UK and TMRCA (Fig. 1).

For Clade I, the strong temporal signal, the basal position of the first hospital outbreak isolate (2015) and ladderised MCC phylogeny suggests the TMRCA of this clade in the UK as 2008 (95% highest posterior density (HPD) interval [1989, 2015]) followed by its spread. The clock rate was defined as 2.764 × 10^−04^. Assessing the introduction of Clade I into the UK placed entry mid-September 2013 (Supplementary Fig. 3; 95% HPD late November 2012 to mid-September 2014 with a posterior probability (PP) of 1). Interestingly, the two resistance-associated *ERG11* point mutations (K143R and Y132F) do not cluster in the phylogeny, suggesting that these SNPs may have originated more than once. Based on the Clade I MCC tree (Fig. 1a), introduction and diversification in the UK began at Royal Brompton Hospital (RBH) and spread to other hospitals, including seeding the later Centre B outbreak that includes the K143R *ERG11* allele. Of note, the branch leading to the Centre B cluster and containing the K143R mutation has a high posterior probability (PP = 0.9202), indicating an introduction to the hospital in 2018 (95% HPD [2017, 2019]); this compares well with the detection of this outbreak in November 2018. Similarly, a group of isolates from one of the two inferred introductions into KCH is also very well supported (PP = 0.9892) with the TMRCA approximately in 2016 (95% HPD [2016, 2017] comparing well with the detection of this outbreak in April 2016^40^.

The topology of the Clade III tree (Fig. 1b) suggests at least two introductions into the UK due to the bifurcating phylogeny and low-quality temporal signal. The long basal branch lengths of the Clade III phylogeny indicate that if a single introduction in 2004 had occurred, it would have remained undetected for ~2–3 years, which is clinically unlikely. The good posterior support of the two sub-clades (Fig. 1b) in late 2006 suggests two introductions of Clade III near-simultaneously, into the UK remains more likely. The clock rate was defined as 3.186 × 10^−04^. Assessing the time-scaled phylogeny placed entry into the UK in late 2014 (Supplementary Fig. 3; 95% HPD early October 2014 to November 2014 with a posterior probability of 1); the earliest Clade III UK isolate (PHE_6) was isolated in 2014, making these dates likely. Only an average of 379 SNPs separate the two Clade III subclades, yet the SNP distribution for each subclade differs; subclade-specific missense variants mapped to 33 and 51 genes unique to subclade A and B, respectively. Whilst no significant corrected gene ontology (GO) terms were associated with the genes in subclade B, the genes in subclade A were significantly overrepresented for the biological process cell wall (1-3)-β-D-glucan biosynthetic process. These differences are consistent with two initial separate introductions of Clade III into the UK, with both subclades introduced into John Radcliffe Hospital and diversification and possible spread to King’s College and Wrexham Park hospitals.

### Inferring direct and indirect patient-to-patient transmission of drug-resistant *C. auris*

Inter- and intra-hospital transmission was analysed using the R package *TransPhylo*. A matrix of the probability of direct transmission for all pairs of individual isolates within each clade was generated to evaluate transmission events within and between hospital centres (Supplementary Fig. 4). We detected 28 (Clade I) and 82 (Clade III) events with a transmission probability of >75%. Clade I events included suggested movements of patients with *C. auris* infection between seven of the eight hospital centres, however two of the transmission events inferred occurred prior to detection indicating either false implied directionality or sampling did not accurately elucidate temporal trends in these data. Clade III transmission events included patients in all three hospital centres; however, 33 of the 82 events were inferred to have occurred prior to the collection date of the baseline isolate.

**Fig. 2:**
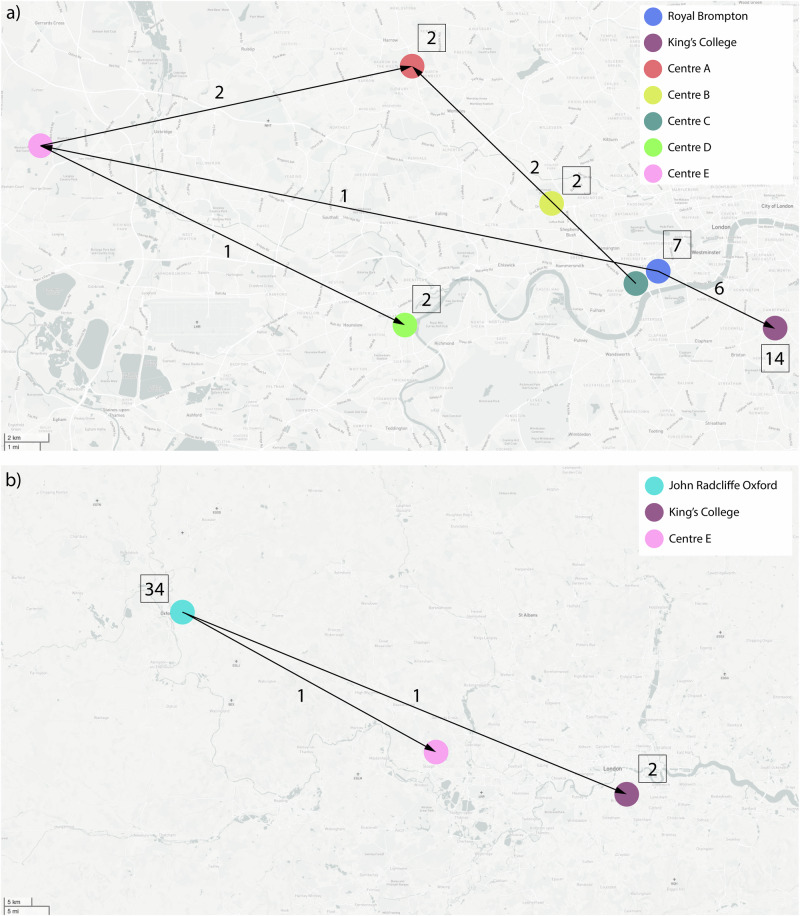
Intra- and Inter-hospital transmission events identified by TransPhylo. **a** Clade I direct transmission directionality according to the analysis in TransPhylo of within hospitals between patients or environmental surfaces and between hospitals. **b** Clade III direct transmission directionality according to the analysis in TransPhylo of within hospitals between patients or environmental surfaces and between hospitals. In both, the numbers above arrows indicate the number of identified inter-hospital transmissions, whilst numbers within squares indicate the number of identified intra-hospital transmissions.

Sixteen transmission events occurred in Clade I within hospitals, while 12 transmission events occurred between hospitals (Fig. 2a, Supplementary Table 3). Twenty-three transmission events in Clade I were robustly supported with a transmission probability of 100%, including 13 within hospital and 10 between hospital events; whilst environmental sampling was not routinely carried out as part of this study, two events also showed links between clinical and environmental isolates (e.g. inanimate surfaces), underscoring the environmental risk that *C. auris* presents in hospital settings. It is, therefore, likely that environmental surfaces play an important role in the transmission of *C. auris* and should not be excluded in future sampling efforts. Of the 13 within-hospital transmission events, 12 were transmission between patients, and one was transmission between a patient and an environmental surface. Based on the patterns of the 12 between hospital events, isolates originated in patients in RBH before being transmitted to King’s College Hospital and Centre C, then later to Centre E and Centre A across London. Of note, the cluster of isolates within Centre B containing the K143R *ERG11* mutation appears to be self-contained and was only found circulating within this hospital.

Eighty transmission events within Clade III occurred within hospitals while two transmission events occurred between hospitals (Fig. 2b, Supplementary Table 4). Sixty transmission events in Clade III had a transmission probability of 100%, including 59 within hospital and one between hospital event; three events also showed movement between clinical and environmental isolates. Of these transmission events, five were multiple isolates within the same patient taken from different sources on different dates. Additional transmission events were between patients and five were transmission events between a patient and an environmental surface. Based on the two intra-hospital transmission events, transmission started in John Radcliffe Hospital and moved to King’s College Hospital and Centre E, which is consistent with the findings of the temporal analysis.

### Microevolution of *C. auris* within a single patient

Twenty-four *C. auris* isolates sequenced as part of this study were taken sequentially from six patients admitted to four London hospitals to investigate microevolution within the human host (Supplementary Table 5). All isolates displayed raised MICs to fluconazole (Supplementary Table 1).

**Fig. 3:**
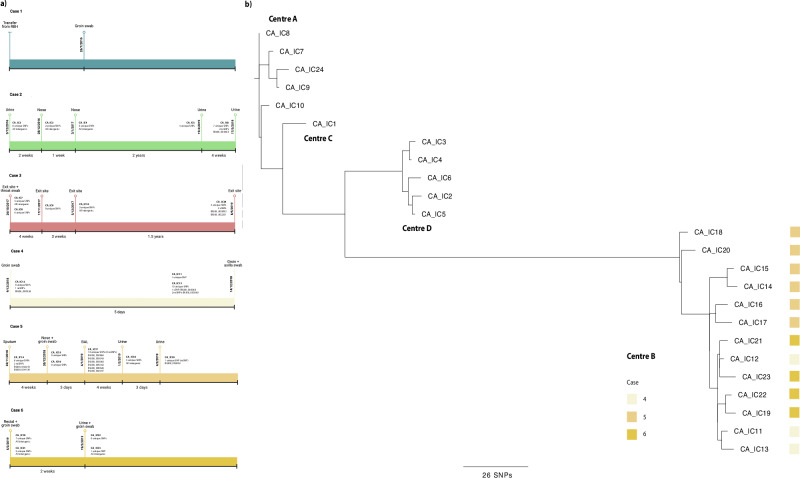
Microevolution of sequential isolates. **a** Timelines of six patients in four London hospitals with sampling dates, location of sample and unique SNPs accumulated. **b** Unrooted, ordered, maximum likelihood phylogenetic tree constructed in RAxML using whole-genome SNPs for sequential isolates taken from six patients in four London hospitals. Branch lengths represent the average number of SNPs.

Five isolates were sequenced from the same patient in Centre D over a period of 29 months (December 2016–May 2019) at multiple bodily locations. Isolates were separated by an average of 8 SNPs, whilst phylogenetic analysis suggests two distinct clusters based on body location with substantial bootstrap support (Supplementary Fig. 5): nose (CA_IC3 and 4), and urine (CA_IC2, 5 and 6). Nose and urine isolates were separated by 3 and 7 SNPs, respectively. Analysing nose and urine isolates separately, only CAIC6 had accumulated non-synonymous SNPs (nsSNPs) when compared to the initial urinary isolate (CA_IC2), with 2 nsSNPs in B9J08_003363 (Fig. 3a), and ncRNA gene tRNA binding lysine. Only intergenic SNPs were found to be unique to either of the two nose isolates (Fig. 3a, Case 2).

Five isolates from a single throat swab and catheter exit sites were sampled from a single patient at Centre A over a period of 18 months (October 2017–May 2019). Despite being collected 18 months after the original four isolates (CA_IC7–10), CA_IC24 is more closely related to CA_IC7–9, separated by only 7 SNPs on average, whilst CA_IC10 is 8 SNPs on average separated from isolates CA_IC7–9 and 24. CA_IC10 potentially represents infection from a genetically distinct *C. auris* variant; transmission analysis via *TransPhylo* corroborates this, identifying a significant intra-hospital transmission of isolates Sample_2 from KCH to Centre A (Supplementary Table 4). Isolates CA_IC7–10 are also identified as significant transmission events, suggesting primary colonisation by a single isolate subsequently colonising the patient. These isolates were compared to the initial throat and exit site swabs taken in October 2017, and only CA_IC24 had unique synonymous SNPs, which mapped to two hypothetical genes, B9J08_002251 and B9J08_005092 (Fig. 3a, Case 3).

Sequential isolates were taken from three other patients (Cases 4–6, Fig. 3a) at Centre B over 4 months. Phylogenetic analysis shows some dissimilarities between isolates from the same patient over time (Fig. 3b): in case 4, the two samples taken on 14 December 2018 (axilla and groyne swabs, CA_IC11 and CA_IC13, respectively) are genetically closely related, separated by only 7 SNPs, yet distinct from the initial groyne swab (CA_IC12) taken 5 days prior. Indeed, CA_IC12 is only separated by 7 SNPs from CA_IC21 and 23 from Case 6. However, given the high number of unique SNPs in each isolate (Fig. 3a), it is probable this patient was infected with at least three genotypes. Similar to Case 2 (Centre D), unique synonymous and nsSNPs were observed in the ncRNA tRNA-Lys gene B9J08_003363 in groyne isolate CA_IC13. The genetically dissimilar isolates from Case 5, six taken from multiple anatomical sites over 3 months, also seem to indicate colonisation by a diverse *C. auris* genetic population: on average isolate were separated by 17 SNPs, yet the two urine isolates (CA_IC18 and CA_IC20, collected one month apart) are only 9 SNPs different, but 23 SNPs apart from other Case 5 isolates. Temporal analysis (Fig. 1) also places these two isolates as basal for Centre B, suggesting that whilst these two isolates were taken last, they potentially represent the original infecting population. More unique SNPs were recovered in this patient, with B9J08_003363 again displaying unique nsSNPs in CA_IC17, isolated from a bronchoalveolar lavage (BAL) fluid sample. Finally, four isolates over a two-week period were collected from Case 6 at Centre B and were on average 9 SNPs apart. The first three isolates (CA_IC19, 21, 22) were groyne and rectal swabs, whereas the final isolate CA_IC23 was from a urine sample. Comparison between early and later isolates yielded only unique SNPs mapping to intergenic regions (Fig. 3a).

## Discussion

*C. auris* poses a severe public health issue globally, causing nosocomial invasive infections in ICUs. We therefore sought to apply cutting-edge genomic approaches, previously developed for elucidating bacterial pathogen dynamics, to over 200 whole-genome sequences and define the temporal and spatial epidemiology of this important pathogen.

Firstly, our study shows that there have been multiple introductions from different spatial origins at different times in the UK. Secondly, this has been followed by within and between-hospital transmission of antifungal drug-resistant isolates. Only Clades I and III, the South Asian and African clades, respectively, were recovered within the UK; this was based on phylogenetic analysis and clade-specific *ERG11* gene mutations from isolates included in this study covering three major UK outbreaks up to 2020, conferring resistance to first-line azole antifungal drugs. Using the whole-genome SNPs, Bayesian inference determined the MRCA of the two clades to be in 2008 for Clade I and 2004 for Clade III. Introduction into the UK based on analyses of these isolates alone was placed in mid-September 2013 and late 2014 for Clades I and III, respectively. A recent study, not conducted on these exact isolates but including a subset of these isolates, placed the introduction of Clade III *C. auris* into the UK as November 2013 (95% HDP: May 2013–April 2014)^44^; the addition of a larger number of isolates could be responsible for differing date estimates. Local-scale transmission patterns within and between hospitals were identified in both clades, showing transmission of *C. auris* from human-to-human and between human and environmental surfaces. Analysis of the microevolution of sequentially collected isolates from single patients in multiple hospitals has shown patients can be colonised and/or infected by a diverse genotype, confirming earlier studies^32^. It is therefore important to collect and sequence multiple *C. auris* per patient, ideally from different body sites, to fully assess the genetic diversity present; whilst differences in drug susceptibility profiles were not observed in sequential isolates in this study, it cannot be ruled out as a possibility, particularly with the use of topical agents at specific body sites, which could negatively impact patient treatment. Interestingly, nsSNPs within B9J08_003363, encoded a ncRNA tRNA-Lys, were identified in multiple isolates from different patients and hospitals (CA_IC6, Centre D; CA_IC13, Case 4 Centre B; CA_IC17, Case 5 Centre B). ncRNAs have been shown to regulate gene expression in response to various conditions, such as antifungal drugs, in what is termed as ‘epimutation’^45,46^. This finding could be explored further to investigate the role of ncRNAs in *C. auris* adaptation within the human host.

The slope of the root-to-tip regression of Clade I and III were found to be 4.901 × 10^−4^ and 3.186 × 10^−4^ substitutions per polymorphic site per year, respectively. These evolutionary rates are reasonably comparable to other epidemiological analyses of *C. auris*, the Royal Brompton outbreak (1.002 × 10^−3^ substitutions per polymorphic site per year) and a global dataset including 304 isolates (1.8695 × 10^−5^ substitutions per polymorphic site per year)^30,32^. The RBH outbreak included the 24 outbreak-specific Clade I isolates and used a relaxed lognormal molecular clock, which accounts for variation in evolution, while the analysis of the global dataset used isolates from all four clades using a strict molecular clock, which assumes a constant rate of evolution. These differences may account for the variability between the two evolutionary rates predicted for *C. auris* in previous studies. A relaxed lognormal molecular clock was also used in this study to account for variation in substitution rates, which has been suggested as an appropriate clock in fungi^47^.

While support for two clades in the UK is well-documented based on hospital-specific outbreak analyses^9,32,48^, this is the first-time temporal analysis on all available Clade I and III WGS data in the UK prior to 2020. According to the Bayesian MCC tree, there was a single introduction with an MRCA in Clade I estimated to be in 2008 (95% HPD interval [1989, 2015]). The Bayesian phylogenetic analysis shows that the first introduction of Clade I into the UK was at RBH, with multiple introductions into the hospital consistent with the conclusions from our original study^32^, due to the scattered isolates throughout the time-scaled phylogeny. This is consistent with the RBH outbreak analysis, which suggested that entry into the hospital was in early 2015 before all other hospitals with Clade I isolates^32^. However, the long branches leading to the isolate on either side of the root have low posterior probabilities (PP = 0.2044), showing poor support for that topology, suggesting these isolates could have been circulating within the hospital before detection. This observation highlights the importance of yeast identification in clinical samples. The introduction of Clade I into the UK does not differentiate between the isolates with the K143R and Y132F point mutations in the *ERG11* gene, despite isolates from Centre B containing the K143R mutation having a very well-supported branch. The MRCA for this hospital is estimated in late 2018 (95% HPD [2017, 2019]), which corresponds to the sample collection dates in the hospital (Supplementary Table 1). Transmission analysis with *TransPhylo* also suggested the Centre B cluster was self-contained. However, there is not sufficient evidence here to confirm this genotype represents a separate introduction into the UK.

The MRCA in Clade III was estimated to be in 2004 (95% HPD interval [1972, 2015]); however, given the bi-clade structure with long basal branches, it is more indicative that the diversity of Clade III has not been fully sampled here. This Clade III isolate was initially introduced into the John Radcliffe Hospital via two introductions before spreading to KCH and Wrexham Park Hospital. This is at odds with the original analysis of Eyre et al. which concluded the outbreak originated from a single introduction from Clade III in 2013^9^. This may be due to differing clock rates. Since the John Radcliffe Hospital only serves 1% of the UK population, two introductions may be likely if a single patient brought in pre-existing diversity or colonisation went undetected or as a tertiary centre for specialised care receiving patients from a wide geography, including internationally. However, the distribution of non-synonymous SNPs in Clade III sub-clade A, which was introduced into the UK slightly earlier than sub-clade B, is significantly overrepresented for the β-D-glucan biosynthetic process, a crucial component of the cell wall. Non-synonymous mutations in genes involved in β-D-glucan biosynthesis could represent an evolution for these isolates to have higher β-D-glucan content in their cell walls. *Aspergillus flavus* isolates with higher β-D-glucan content may show amphotericin B resistance^49^; further analysis into cell wall content needs to be carried out to confirm the difference in Clade III isolates within the UK.

As nosocomial transmission of *C. auris* has been highlighted as such an important factor in healthcare-associated infection control measures, reconstructing significant transmission events from genomic data can be informative. We applied *TransPhylo*^50^, a software tool implemented as an R package, to link the *C. auris* phylogenies and predicted transmission events, and identified 28 and 82 individual transmission events for Clade I and III, respectively, based on the 75% probability threshold of direct transmission of isolates (Fig. 2). Since *TransPhylo* was intended to be applied to bacterial pathogens, which can have clonal genomes, *C. auris* was deemed to be a logical dataset for application of this software. In Clade I, 16 of the events occurred within hospitals, while 12 occurred between hospitals (Supplementary Table 3). Between-hospital patterns of transmission show that the first observed occurrence of Clade I in the UK started in RBH, confirming that this is the European index outbreak for *C. auris*. The transmission analysis was also able to identify the Centre B isolates with the K143R point mutation in the *ERG11* gene as a separate transmission chain circulating only within the hospital. TransPhylo’s built-in algorithm to identify introductions into a population of interest^51^ allowed the identification of the separate transmission chain and unsampled intermediates for the different Clade I *ERG11* point mutations. By comparing the transmission events in Clade III to the corresponding MCC phylogeny, we showed that all transmission events stayed within their corresponding location on either side of the MCC root (Supplementary Table 4). Eighty of the 82 events occurred within hospitals, with four genotypes accounting for 79.3% of all transmission events for these isolates. Two inter-hospital transmissions showed patient/isolate movement from John Radcliffe to both KCH and Centre E. Analysis from the John Radcliffe outbreak confirmed that the isolates from temperature probes (environmental surface) were scattered throughout the phylogeny, and there was no evidence of transmission from patient-to-patient in nearby beds^9^; here, only four of the 82 transmission events were shown to occur between the temperature probes and patients, although this is likely a result of under-sampling of temperature probes early in the outbreak. Earlier analyses of this outbreak using WGS failed to find a relationship between patient bed proximity and genomic distances, which may be an artefact of having only a few environmental samples obtained later in the outbreak. Adding patient admission and/or discharge data to these inferred transmissions would be powerful, and we recommend future sampling be accompanied by these data.

A common limitation that confounds the inference of transmission events here is that the sample collection date of the second isolate occurs before the collection date of the primary isolate. There are multiple reasons why this could transpire. It is possible that the sample size for each of the clades was not large enough to evaluate due to missing intermediate isolates on the pathway to transmission. The movement of hospital equipment, staff, and patients contaminated with *C. auris* from hospital to hospital could affect the sample collection dates and transmission between hospitals; for example, if a patient with chronic undetected asymptomatic *C. auris* contaminated a piece of equipment that was then used on an independent patient who then developed symptomatic infection, the sample collection date for the second patient in this example would be prior to the first patient who would not be identified with *C. auris* until after they show symptoms, are detected by contact tracing or possibly never. Also, the amount of community carriage is difficult, or impossible, to assess, and community carriage of *C. auris* to date is unknown. It is likely there remains a significant lack of detection of colonised patients in certain settings. Finally, *C. auris* is incredibly resilient, often showing resistance to disinfectants and can form biofilms, allowing it to remain dormant and persist on surfaces for long durations, possibly allowing patients to be infected by the same isolate months apart, although this could also result from undetected chains of transmission^9,23,29,32^.

The present analysis evaluated the spatial and temporal patterns of Clade I and III isolates of *C. auris* within the UK. Future analyses would benefit from comparing these isolates with the full global dataset to help expand understanding of where the UK fits within the global phylogenetic tree. By including within-clade introductions and differentiation between the *ERG11* mutations, we could also understand the temporal origins of outbreaks and infections with Y132F and K143R mutations in the *ERG11* gene. In addition, the use of patient, equipment and staff records, after ethics approval is granted, to validate the outputs from TransPhylo can be used to demonstrate the capability of identifying transmission patterns retrospectively. If accurate, TransPhylo can also be utilised to predict local-transmission dynamics prospectively in the beginning of outbreaks to help in the earlier implementation of infection prevention and control measures and containment of the outbreak.

This study assesses the temporal and spatial dynamics of *C. auris* in terms of genomic epidemiology, using data from the UK. Leveraging the power of whole genome sequencing, we identified at least three introductions into the UK and, importantly, providing insights into inter- and intra-hospital transmission of *C. auris*. These analyses underscore the need to utilise the vast numbers of genome sequences available for *C. auris* to further assess transmission routes into and between hospitals and help to inform hospital policy on outbreak management.

## Methods

### Sample preparation, sequencing and data acquisition

Twenty-four isolates were collected from six patients in four London hospitals, covering an area of ~54 km^2^ (isolate details in minimum inhibitory concentration (MIC) antifungal susceptibility data in Supplementary Table 1) between 2016 and 2019. In addition, 43 isolates from seven centres across the UK were collected between 2013 and 2016. Isolates were confirmed as *C. auris* using matrix-assisted laser desorption/ionisation-time of flight (MALDI-TOF) mass spectrometry (MS). High-quality, high molecular weight genomic DNA was extracted from the isolates using the MasterPure yeast DNA extraction kit (EpiCentre) and sent to the Sanger Institute (Wellcome Genome Campus, Cambridge, UK) for the construction of DNA libraries and sequencing. All isolates were sequenced using TruSeq Nano library preparation, and Illumina HiSeq2500 sequencing of 2 × 250 bp paired-end reads at The Centre for Genomic Pathogen Surveillance (Wellcome Genome Campus, Cambridge, UK).

Publicly available whole genome sequences from an additional 140 isolates from across the UK were combined with these 67 newly sequenced isolates. Isolates were collected from clinical and contaminated environmental or equipment sources from nine hospitals between June 2014 and May 2019 in the UK. The samples were sequenced using either the Illumina HiSeq 2500 platform using the HiSeq Rapid SBS Kit v2 500-cycles (Illumina) or the MiSeq platform (Illumina) using the MiSeq Reagent Kit v2 500-cycles (Illumina). All isolates included in this study are summarised in Supplementary Table 1.

### Antifungal susceptibility testing

Antifungal susceptibility testing was completed for the majority of isolates using the CLSI broth microdilution method M27-A3^52^. For a subcollection of tested isolates, the susceptibility testing was carried out using Sensititre YeastOne YST-10 broth dilution panels (Thermo Fisher Scientific, UK) according to the manufacturer’s instructions. The susceptibility of some isolates was determined according to the standard EUCAST method^53^. MICs for isolates tested are reported in Supplementary Table 1.

### Bioinformatic analysis

Analysis was first performed for the whole UK dataset then clade-specific analyses performed separately. All isolates were initially aligned to the reference genome of B8441 from Pakistan (GenBank accession PEKT00000000.2^5^). The data showed there were at least two introductions to the UK from two different clades, Clade I and Clade III, thus for analysis and molecular dating, the B8441 reference (GCA_002759435) was used for alignment of Clade I and reference genome B11221 (GenBank accession PGLS00000000.1^5^) from South Africa was used for alignment of Clade III isolates using Burrows-Wheeler Aligner (BWA) v0.7.17 mem^54^ and converted to sorted BAM format using SAMtools v1.16.1^55^. Duplicate reads were identified and marked using Picard v2.27.4^56^. Base Quality Score Recalibration (BQSR) was performed on all data.

Genome Analysis Toolkit (GATK) v4.2.6.1^57^ ‘HaplotypeCaller’ was used to call single nucleotide polymorphisms (SNPs) excluding repeat regions in the genome (identified via RepeatMasker v4.0.6). SNPs were labelled as low confidence if they met at least one of the following parameters: DP < 5, GQ < 50, MQ < 40, MQRankSum < −12.5, ReadPosRankSum < −8.0, SOR > 4.0, or were not present in at least 90% of reads.

SNPs were converted to presence/absence data with respect to reference. Low-confidence SNPs were classed as missing. Maximum likelihood (ML) phylogenies were constructed using RAxML v8.2.9^58^ rapid bootstrap analysis over 5000 iterations using the BINCAT model of rate heterogeneity. The phylogeny of sequential isolates was constructed as above but with 1000 bootstrap iterations using the GTRGAMMA model of rate heterogeneity. Phylogenetic trees were visualised in FigTree v1.4.4. SNPs were annotated using snpEff v5.1^59^ and summarised for *ERG11*.

### Molecular dating of *C. auris* isolates

Bayesian dating inferences and phylogenetic reconstruction were performed using BEAST v2.6.3 on whole nuclear genome alignments of 114 Clade I isolates, and 93 Clade III isolates with recorded sampling dates. Due to differences in the sizes of Clade I and III *C. auris* genomes, the two clades were analysed separately. TempEst v1.5.3^60^ was used to identify significant root-to-tip distances at each node across the phylogeny. Root-to-tip regression indicating the presence of a molecular clock was identified. Therefore, to quantify the clock rates for the UK Clade I and Clade III isolates separately, Bayesian phylogenies were designed in BEAUti2 for analysis under a relaxed log-normal molecular clock with a Generalised Time Reversible (GTR) nucleotide substitution model in BEAST2 v2.7.5. The specimen collection dates were used as the tip dates of the isolates and were imported days before the present. A relaxed log-normal molecular clock was chosen to account for variation in substitution rate between lineages. Bayesian Markov chain Monte Carlo (MCMC) analyses were run for 50 million steps to reach convergence in the posterior distributions (confirmed using Tracer v1.7.1) with a logging frequency of 5000 runs and burn-in rate of 10%. Tree topology was summarised by generating a clade credibility tree in TreeAnnotator v2.6.3 with 10% burn-in and node heights set to common ancestor heights. The phylogenies were visualised in FigTree v1.4.4.

To assess the timing of entry of Clades I and III into the UK, representatives of each outbreak were assessed along with Clade I isolates B11096 and B11210 (isolated in 2014 and 2013, respectively) and Clade III isolate B11221 (isolated in 2013)^5^. A HKY substitution model with optimised relaxed clock model was used, with a calibrated yule model as the tree prior. A calibration node containing UK outbreak isolates (i.e. excluding B11096, B11210, and B11221) was also included. MCMC analyses were set and analysed as above.

### Outbreak transmission analysis

The R package *TransPhylo*^51^ v1.4.4 uses a phylogenetic tree to infer the order and direction of transmission between isolates while taking into account within host genetic diversity and mutations common to the isolates. *TransPhylo* was used to examine the transmission of isolates within each clade within the UK. MCMC analysis was run for 10,000 iterations with shape and scale parameters for the gamma distribution representing generation time and time at which observations of cases stopped. The parameters for the gamma distribution were set to 10 and 1, respectively, as a generation time of 10 days was assumed to be plausible in an inpatient setting (based on personal communication with X. Didelot). Convergence was assessed by plotting the tree output and the “CODA” package^61^ in R version 4.0.4. Transmission trees and probability of direct transmission for all pairs of individuals were plotted in R version 4.0.4.

## Supplementary information

Supplementary information

## Data Availability

All novel raw reads in this study have been submitted to the European Nucleotide Archive under project accession PRJEB36822.

## Code availability

All code is provided at https://github.com/mycologenomics.
