# Supplementary material for: Genomic epidemiology describes introduction and outbreaks of antifungal drug-resistant Candida auris
Source: NPJ Antimicrob Resist. 2024 Sep 30;2:26. doi: 10.1038/s44259-024-00043-6 (PMC11442302; doi:10.1038/s44259-024-00043-6)
Supplement: Supplementary file 1 — Supplementary information [file 44259_2024_43_MOESM1_ESM.pdf]

Supplementary Figure 1 – Regression plots a) Clade I and **b)** Clade III

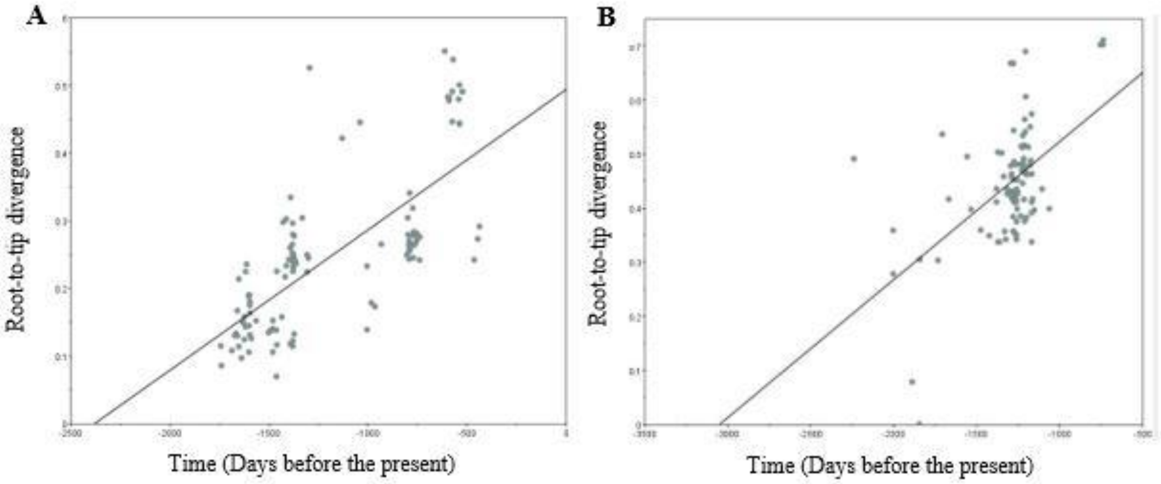

Supplementary Figure 2 – Maximum likelihood phylogeny of all 207 isolates included within this study

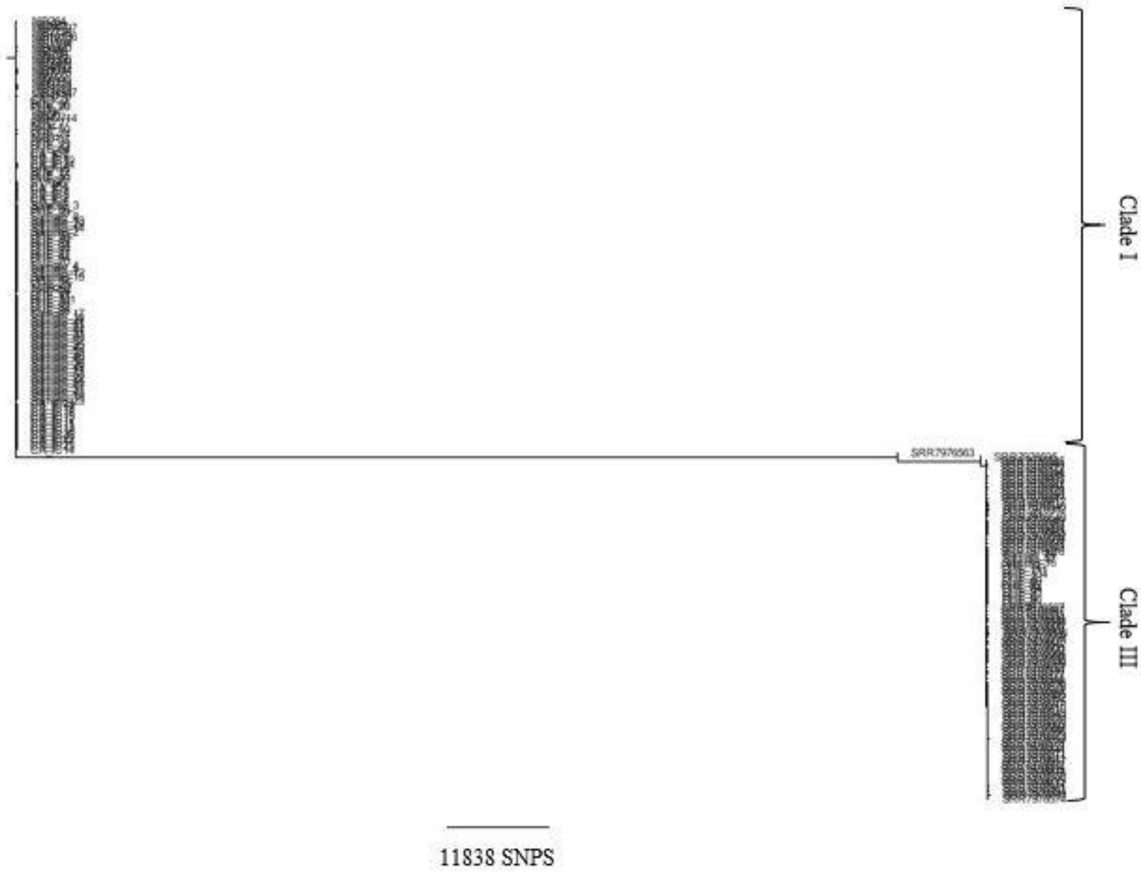

Supplementary Figure 3 – Time-scale maximum-clade credibility phylogeny of a) Clade I UK isolates, and b) Clade III UK isolates, plus additional non-UK isolates to assess the possible timing of the introduction into the UK. Posterior distributions shown as node bar to demonstrate confidence

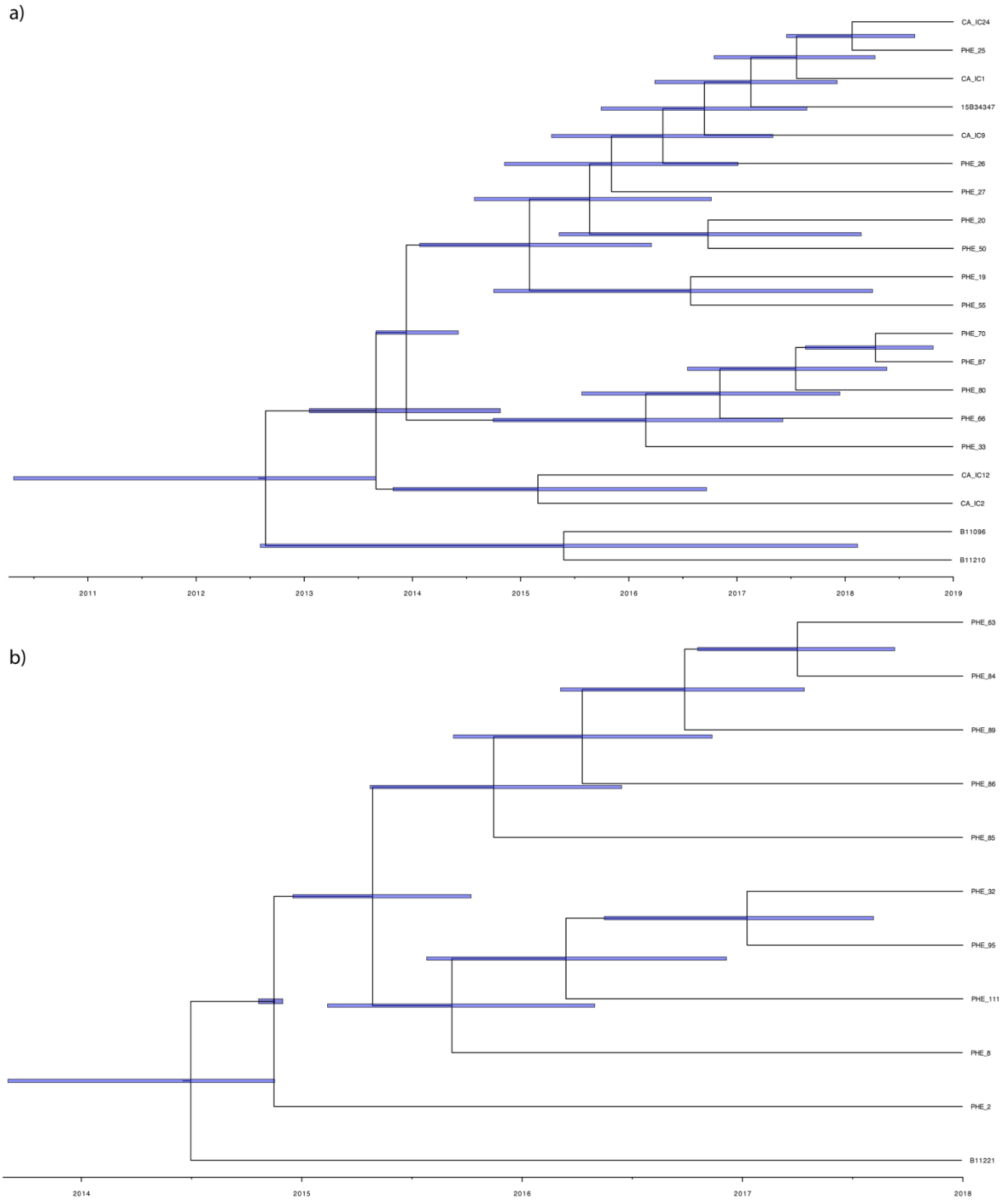

Supplementary Figure 4 - Matrices of probability of direct transmission for all pairs of individual isolates within Clade I (a) and III (b). These matrices show the probability of direct transmission between isolates with no probability in dark pink and 100% probability in turquoise. Sample names were removed**.** Tables 3 (Clade I) and 4 (Clade III) in the appendix include information on transmission patterns including probability, collection dates, hospitals, sources and patient numbers.

a)

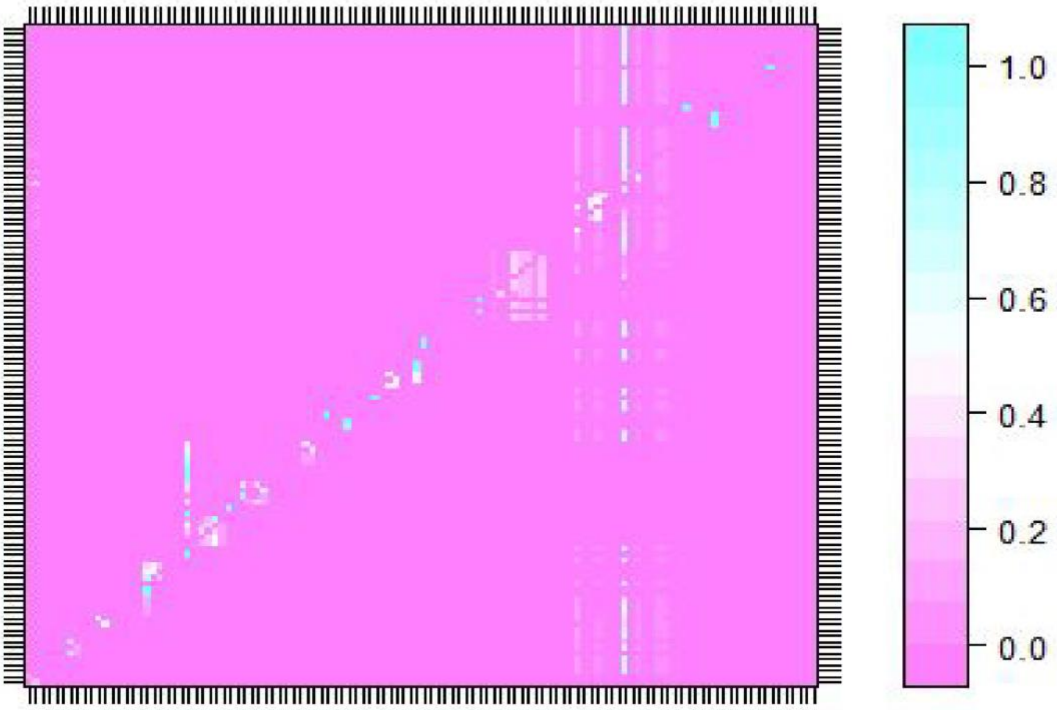

b)

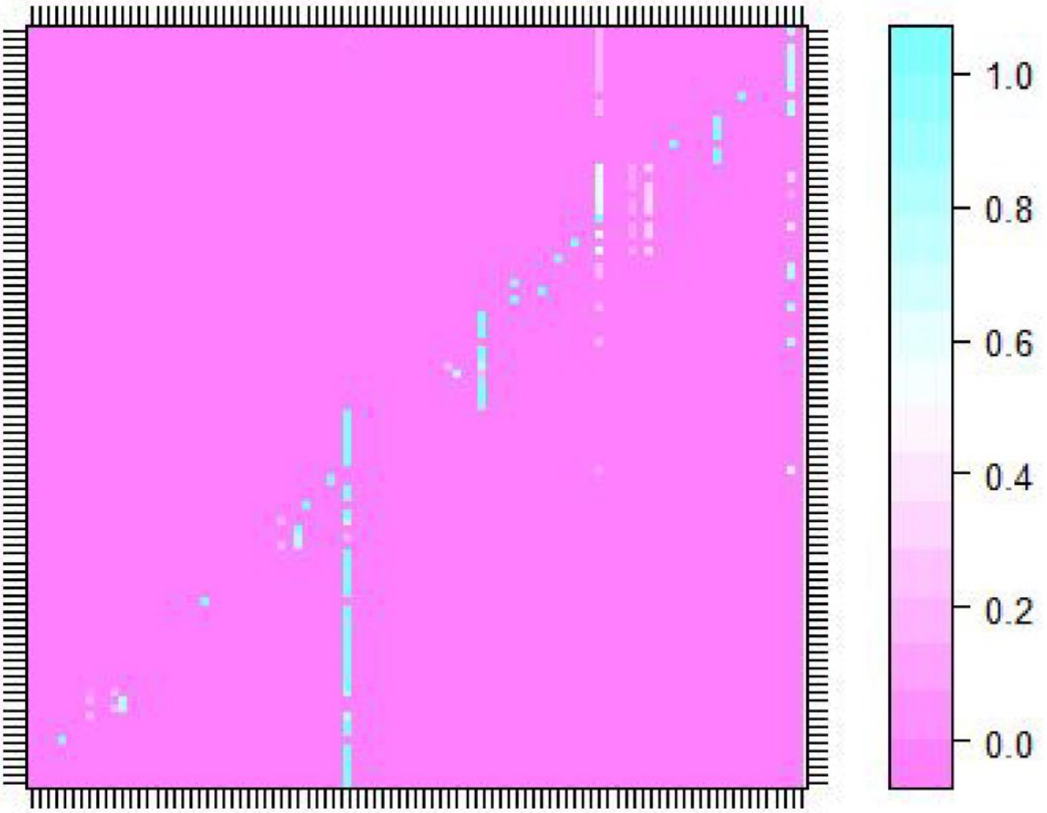

Supplementary Figure 5 – Maximum likelihood phylogeny of 24 isolates from 4 London hospitals, including sequential isolates from single patients. Bootstrap iterations over 1000 replicates performed on WGS SNPs data, where branch lengths represent average number of SNPs. Bootstrap support below 75% indicated on branches.

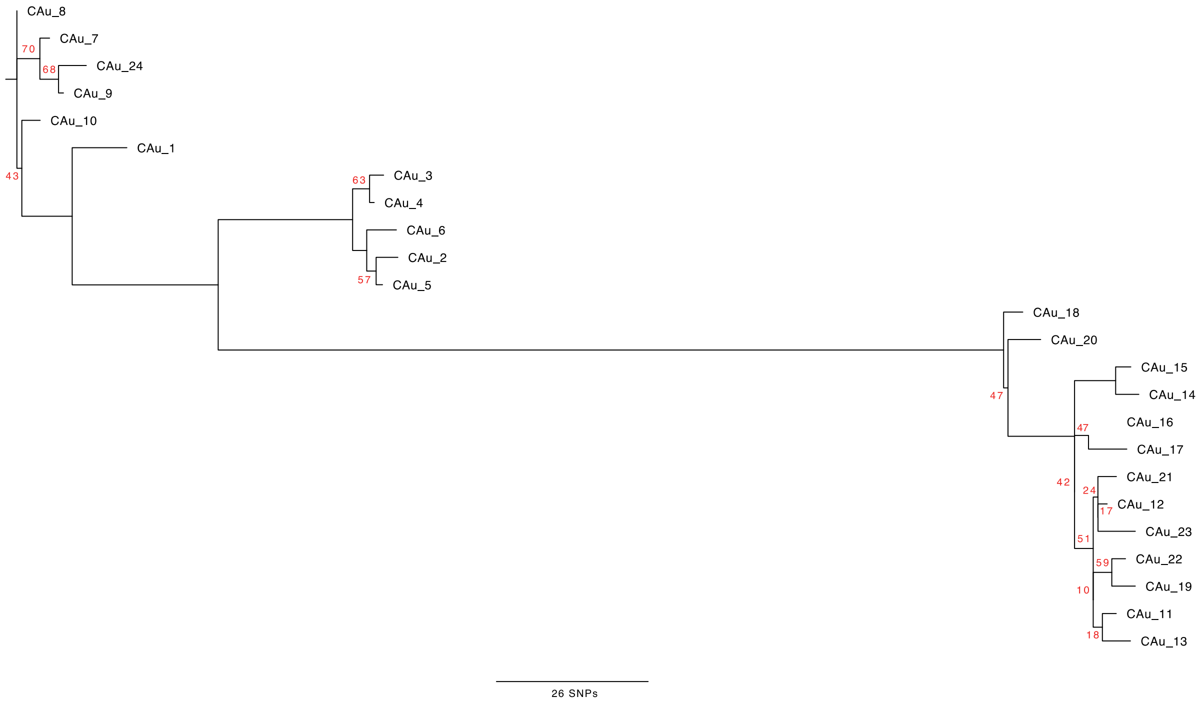

Supplementary Table 1 – *C. auris* isolates included in this study, summarised by Clade and hospital centre, with project accession number for data repository. *ERG11* mutations and minimum inhibitory concentrations (MICs) also provided: FLC=fluconazole; ITR=itraconazole; VRC=voriconazole; POS=posaconazole; CAS=caspofungin; AMB=amphotericin B; FLY=5-flucytosine; blank=not done/available.

Clade	Hospital Centre	Isolate	Sample Collection Date	*ERG11* mutation	FLC	ITR	VRC	POS	CAS	AMB	FLY	Discriminant Analysis of Principal Components (DAPC) Cluster Number
I	Centre C (PRJEB36822)	CA_ICA1	26/07/2016	Y132F	16	<0.03	0.03	0.008	0.125	0.5	<0.06	1
	Centre B (PRJEB36822)	CA_IC11	14/12/2018	K143R	128	0.06	0.125	0.03	0.06	0.5	<0.06	1
		CA_IC12	09/12/2018	K143R	>64	0.125	1	0.125	0.5	0.5	0.25	1
		CA_IC13	14/12/2018	K143R								1
		CA_IC14	22/11/2018	K143R	128	0.125	0.125	0.06	0.125	1	<0.06	1
		CA_IC15	30/12/2018	K143R	128	0.06	0.125	0.03	0.06	0.5	<0.06	1
		CA_IC16	30/12/2018	K143R	64	0.06	0.125	0.06	0.06	0.5	<0.06	1
		CA_IC17	04/01/2019	K143R	128	0.06	0.125	0.03	0.125	0.5	<0.06	1
		CA_IC18	01/02/2019	K143R	8	<0.03	<0.008	<0.008	0.125	2	<0.06	1
		CA_IC19	05/02/2019	K143R	>64	0.25	1	0.25	0.5	1	0.25	3
		CA_IC20	04/02/2019	K143R	8	<0.03	0.015	<0.008	0.125	1	<0.06	1
		CA_IC21	05/02/2019	K143R								1
		CA_IC22	19/02/2019	K143R								1
		CA_IC23	19/02/2019	K143R								1
	King's College (PRJEB36563)	PHE_101	25/10/2016	Y132F	16		0.125			2	<0.125	3
		PHE_33	28/07/2016	Y132F	32		0.125			2	8	3
		PHE_64	29/08/2016	Y132F	64					1	4	3

		PHE_66	08/09/2016	Y132F	>64		0.5		0.125	1	>64	3
		PHE_70	14/09/2016	Y132F		0.25	0.5	0.06	1.5	1	4	3
		PHE_80	27/09/2016	Y132F	64		0.25		>32	0.25	1	3
		PHE_81	03/10/2016	Y132F	32		0.125		0.125	1	>64	3
		PHE_87	16/10/2016	Y132F	64		0.5		0.25	1	32	3
		PHE_88	19/10/2016	Y132F	32		0.5			1		3
		PHE_91	30/10/2016	Y132F	32		0.25		0.125	1	0.25	3
		PHE_92	16/10/2016	Y132F	32	<0.03	0.125	<0.03	0.5	1	0.125	3
		PHE_93	20/10/2016	Y132F	>64		0.25		0.125	1	32	3
		PHE_97	17/10/2016	Y132F	64		0.5		0.19	1	16	3
		PHE_98	24/10/2016	Y132F	>64		0.25			2	4	3
		PHE_99	14/10/2016	Y132F	32		0.125			2	0.25	3
		Sample_10	09/01/2017	Y132F	>64		2			1	8	2
		Sample_11	06/01/2018	Y132F	32		0.5			1		3
		Sample_12	21/09/2017	Y132F	>64		>16		1	1	8	6
		Sample_13	28/12/2016	Y132F								5
		Sample_14	14/09/2016	Y132F								5
		Sample_17	18/07/2018	Y132F	32	0.06	1	0.06		1	0.125	3
		Sample_18	20/07/2018	Y132F	32	0.03	0.125	0.03	0.5	1	0.125	3
		Sample_2	15/10/2016	Y132F								5
		Sample_20	17/10/2016	Y132F								3
		Sample_21	14/10/2016	Y132F								3
		Sample_22	22/06/2018	Y132F	32		0.25			1		3
		Sample_23	07/06/2018	Y132F	32		0.25			1		3
		Sample_3	24/10/2016	Y132F								3
		Sample_30	25/05/2018	Y132F	32	0.03	0.25	0.03	0.5	1	0.125	3
		Sample_35	30/05/2018	Y132F					0.25	1	0.5	5
		Sample_36	14/06/2018	Y132F								5

	Sample_37	18/06/2018	Y132F	64		0.25			1		5
	Sample_38	16/06/2018	Y132F								3
	Sample_4	06/10/2016	Y132F								6
	Sample_41	27/05/2018	Y132F								3
	Sample_42	15/05/2018	Y132F								5
	Sample_43	27/05/2018	Y132F					0.25	1	0.125	5
	Sample_44	01/06/2018	Y132F					0.25	1	0.25	5
	Sample_45	26/05/2018	Y132F					0.25	1	0.25	5
	Sample_46	28/05/2018	Y132F					0.25	1	0.125	5
	Sample_47	31/05/2018	Y132F					0.5	1	0.125	5
	Sample_48	29/05/2018	Y132F								5
	Sample_49	20/05/2018	Y132F					0.25	1	0.25	5
	Sample_5	22/06/2017	Y132F								2
	Sample_50	21/05/2018	Y132F								5
	Sample_52	03/07/2018	Y132F								5
	Sample_53	03/07/2018	Y132F								5
	Sample_55	03/07/2018	Y132F								5
	Sample_8	01/11/2016	Y132F								3
Centre A	CA_IC10	05/12/2017	Y132F	16	<0.03	0.03	<0.008	0.06	0.25	<0.06	3
(PRJEB36822)	CA_IC24	08/05/2019	Y132F	16	<0.03	0.03	<0.008	0.125	0.5	<0.06	1
	CA_IC7	26/10/2017	Y132F								1
	CA_IC8	26/10/2017	Y132F	16	<0.03	0.125	<0.03	0.5	1	<0.125	1
	CA_IC9	17/11/2017	Y132F	32	<0.03	0.03	<0.008	0.125	0.5	<0.06	1
Royal	15B6	22/10/2015	Y132F	256	0.06						5
Brompton	15B10	28/12/2015	Y132F	256	16						3
(PRJEB26393,	15B5	19/10/2015	Y132F	256	16						3
with PHE_	16B27a	15/03/2016	Y132F	256	16						5
isolates	16B13	11/01/2016	Y132F	256	0.06						5

	submitted	16B12	04/01/2016	Y132F	256	16						5
	under	16B16	21/02/2016	Y132F	256	0.03						4
	PRJEB36822)	16B30	17/10/2016	Y132F	128	0.06						3
		16B31	16/10/2016	Y132F	256	0.06						3
		16B18	01/02/2016	Y132F	256	0.12						5
		16B15a	06/02/2016	Y132F	16	0.015						5
		16B20	16/02/2016	Y132F	256	16						3
		16B21	22/02/2016	Y132F	16	0.03						5
		16B22a	27/02/2016	Y132F		0.03						3
		16B24b	07/03/2016	Y132F		2						3
		16B22b	09/03/2016	Y132F	256	2						3
		16B15b	09/03/2016	Y132F	8	0.03						2
		16B24a	12/03/2016	Y132F	256	2						5
		16B25	13/03/2016	Y132F	64	0.03						3
		16B26	14/03/2016	Y132F	256	16						3
		16I30	21/03/2016	Y132F	256	16						3
		16I29a	18/01/2016	Y132F								3
		16I29b	18/01/2016	Y132F								5
		16I33	16/06/2016	Y132F	128	0.03						3
		16I34	23/10/2016	Y132F	256	0.03						3
		16I17	08/02/2016	Y132F								5
		16I27b	14/03/2016	Y132F								4
		PHE_20	08/07/2016	Y132F	>64	16	>16	>16		1	>64	3
		PHE_23	08/07/2016	Y132F	16	<0.03	0.125	<0.03		1	<0.125	3
		PHE_24	08/07/2016	Y132F								3
		PHE_25	08/07/2016	Y132F								2
		PHE_28	09/03/2016	Y132F								2
		PHE_34	26/07/2016	Y132F	8	0.03	0.25	0.03		1	>64	3

	PHE_35	28/07/2016	Y132F							<0.125	3
	PHE_50	14/04/2016	Y132F								3
Centre G	PHE_26	14/12/2015	Y132F	32	<0.03	0.06	<0.03		2	<0.125	3
(PRJEB36822)	PHE_27	15/02/2016	Y132F	16	<0.03	0.06	<0.03		1	<0.125	3

	Centre D (PRJEB36822)	CA_IC2	03/12/2016	Y132F	64	<0.03	0.125	<0.008	0.125	1	<0.06	1
		CA_IC3	28/12/2016	Y132F	64	<0.03	0.125	<0.008	0.125	1	<0.06	1
		CA_IC4	03/01/2017	Y132F								1
		CA_IC5	19/04/2019	Y132F								1
		CA_IC6	17/05/2019	Y132F								1
	Centre E (PRJEB36822)	PHE_12	11/01/2016	Y132F	16	<0.03	0.125	<0.03		1	<0.125	3
		PHE_19	24/06/2016	Y132F	16	<0.03	0.125	<0.03		1	<0.125	3
		PHE_55	22/08/2016	Y132F								3
		PHE_82	05/10/2016	Y132F	16	0.06	0.5		0.06	0.5		3
III	John Radcliffe (Project Accession PRJ415955, with PHE_ isolates submitted under PRJEB36822)	PHE_104	25/10/2016	F126L	>64	0.5	4	0.25		1	0.25	1
		PHE_111	02/11/2016	F126L	>64	0.5	8	0.125		1	0.25	1
		PHE_2	27/05/2015	F126L	>64	0.25	1	0.06		4	<0.125	1
		PHE_32	22/04/2016	F126L	>64	0.25	4	0.06		1	0.5	1
		PHE_8	02/02/2015	F126L	>64	0.25	2	0.06		1	<0.125	1
		PHE_84	16/10/2016	F126L	>64	0.25	2	0.06		1	1	1
		PHE_85	29/10/2015	F126L	>64	0.5	4	0.06		2	<0.125	1
		PHE_86	13/07/2016	F126L	>64	0.25	2	0.06		1	<0.125	1
		PHE_95	16/10/2016	F126L	>64	0.5	4	0.06		2	0.25	1
		SRR7976540	10/02/2017	F126L								5
		SRR7976541	05/05/2017	F126L								1
		SRR7976542	17/11/2016	F126L								1
		SRR7976543	15/01/2017	F126L								5
		SRR7976544	03/04/2017	F126L								2
		SRR7976545	25/01/2017	F126L								5
		SRR7976546	13/02/2017	F126L								1
		SRR7976547	30/01/2017	F126L								5
		SRR7976548	30/08/2017	F126L								5
		SRR7976549	02/02/2015	F126L								5

		SRR7976550	19/12/2016	F126L								1
		SRR7976551	20/02/2017	F126L								5
		SRR7976552	17/02/2017	F126L								1
		SRR7976553	18/03/2017	F126L								5
		SRR7976554	10/02/2017	F126L								5
		SRR7976555	30/01/2017	F126L								5
		SRR7976556	22/05/2017	F126L								5
		SRR7976557	25/10/2016	F126L								1
		SRR7976558	07/04/2017	F126L								3
		SRR7976559	22/03/2017	F126L								5
		SRR7976560	16/05/2017	F126L								1
		SRR7976561	10/05/2017	F126L								1
		SRR7976562	14/04/2017	F126L								5
		SRR7976563	16/01/2017	F126L								6
		SRR7976564	10/01/2017	F126L								5
		SRR7976565	27/03/2017	F126L								5
		SRR7976566	24/01/2017	F126L								5
		SRR7976567	31/05/2017	F126L								1
		SRR7976568	30/01/2017	F126L								5
		SRR7976569	15/02/2017	F126L								6
		SRR7976570	29/01/2017	F126L								1
		SRR7976571	16/05/2017	F126L								5
		SRR7976572	10/03/2017	F126L								5
		SRR7976573	15/01/2017	F126L								1
		SRR7976574	09/01/2017	F126L								5
		SRR7976575	05/04/2017	F126L								5
		SRR7976576	07/05/2017	F126L								5
		SRR7976577	19/12/2016	F126L								2

		SRR7976578	27/01/2017	F126L								1
		SRR7976579	10/07/2015	F126L								1
		SRR7976580	26/04/2017	F126L								2
		SRR7976581	20/02/2017	F126L								1
		SRR7976582	24/11/2015	F126L								1
		SRR7976583	04/04/2017	F126L								5
		SRR7976584	28/11/2016	F126L								5
		SRR7976585	03/04/2017	F126L								5
		SRR7976586	15/01/2017	F126L								5
		SRR7976587	13/02/2017	F126L								1
		SRR7976588	08/03/2017	F126L								3
		SRR7976589	17/02/2017	F126L								1
		SRR7976590	15/03/2017	F126L								3
		SRR7976591	11/12/2016	F126L								1
		SRR7976592	24/03/2017	F126L								4
		SRR7976593	04/04/2017	F126L								1
		SRR7976594	30/01/2017	F126L								5
		SRR7976595	17/07/2017	F126L								4
		SRR7976596	17/05/2017	F126L								5
		SRR7976597	05/12/2016	F126L								1
		SRR7976598	09/04/2017	F126L								5
		SRR7976599	25/01/2017	F126L								5
		SRR7976600	29/03/2017	F126L								5
		SRR7976601	16/05/2017	F126L								4
		SRR7976602	16/05/2017	F126L								1
		SRR7976603	24/04/2017	F126L								1
		SRR7976604	09/01/2017	F126L								1
		SRR7976605	08/02/2017	F126L								5

		SRR7976606	30/01/2017	F126L								1
		SRR7976607	10/04/2017	F126L								1
		SRR7976608	13/02/2017	F126L								1
		SRR7976609	29/01/2017	F126L								1
		SRR7976610	10/04/2017	F126L								5
		SRR7976611	13/02/2017	F126L								5
		SRR7976612	13/02/2017	F126L								5
		SRR7976613	25/01/2017	F126L								2
		SRR7976614	02/01/2016	F126L								1
		SRR7976615	31/03/2017	F126L								5
		SRR7976616	13/07/2015	F126L								1
		SRR7976617	22/03/2017	F126L								5
	King's College (Project Accession PRJEB36563)	Sample_16	18/07/2018	F126L	64		2			1		1
		Sample_24	21/07/2018	F126L	64		1			0.5		1
		Sample_57	04/07/2018	F126L	64		2			1		1
	Centre E (PRJEB36822)	PHE_63	02/09/2016	F126L	>64		4		0.25	0.25		1
		PHE_89	16/05/2016	F126L								1
	HCA laboratories (PRJEB36822)	PHE_6	10/06/2014	F126L	>64	1	4	0.125		1	<0.125	1

Supplementary Table 2 – Average (mean), median (in brackets) and range of single nucleotide polymorphism (SNP) distance between isolates in individual hospital centres and within each clade. Hospital centres with no isolates for a particular clade are denoted as '-'.

	**Clade average**	**Centre C**	**Centre B**	**John Radcliffe**	**King's College**	**Centre A**	**Royal Brompton**	**Centre G**	**Centre D**	**Centre E**
Clade I	213 (178) Range: 2-1147	Only 1 isolate in hospital	69 (75) Range: 25-133	-	127 (174) Range: 2-565	78 (97) Range: 45-141	96 (149) Range: 4-313	25 Only two isolates	71 (58) Range: 32-92	120 (135) Range: 29-205
Clade III	151 (123) Range: 0-977	-	-	139 (123) Range: 0-734	94 (75) Range: 0-197	-	-	-	-	22 (22) Only two isolates

Supplementary Table 3: Transmission between Clade I isolates using *TransPhylo* with a probability threshold above 75%. The transmission pathway is from isolate 1 to isolate 2 including probability of transmission from isolates, date of isolate collection, hospital, source and patient numbers. Patient numbers correspond to what was provided by each hospital centre.

Transmission -->											
Isolate 1	Isolate 2	Probability of Transmission from Isolate 1 to Isolate 2	Date of Collection Isolate 1	Date of Collection Isolate 2	Hospital Isolate 1	Hospital Isolate 2	Source Isolate 1	Source Isolate 2	Patient Number Isolate 1	Patient Number Isolate 2	Notes
16B9044	16B9260	1	07/03/2016	09/03/2016	Royal Brompton	Royal Brompton	Clinical	Clinical	Patient C	Patient A	
16I10532	16B6373	1	21/03/2016	16/02/2016	Royal Brompton	Royal Brompton	Clinical	Clinical	Patient B	Unknown	Collection Date of Isolate 2 before Isolate 1
16I10532	16B7775	1	21/03/2016	27/02/2016	Royal Brompton	Royal Brompton	Clinical	Clinical	Patient B	Patient A	Collection Date of Isolate 2 before Isolate 1
CA_IC7	CA_IC8	1	26/10/2017	26/10/2017	Centre A	Centre A	Clinical	Clinical	3	3	Same collection date, same patient
PHE_19	PHE_50	1	24/06/2016	14/04/2016	Centre E	Royal Brompton	Clinical	Clinical	Unknown	Unknown	Collection Date of Isolate 2 before Isolate 1
Sample_13	Sample_53	1	28/12/2016	03/07/2018	King’s College	King’s College	Clinical	Environment	24	N/A	
Sample_36	Sample_45	1	14/06/2018	26/05/2018	King’s College	King’s College	Environment	Clinical	N/A	39	Collection Date of Isolate 2 before Isolate 1
Sample_42	Sample_46	1	15/05/2018	28/05/2018	King’s College	King’s College	Clinical	Clinical	37	42	
Sample_48	Sample_44	1	29/05/2018	01/06/2018	King’s College	King’s College	Clinical	Clinical	40	45	

Sample_50	Sample_43	1	21/05/2018	27/05/2018	King's College	King's College	Clinical	Clinical	31	38	
Sample_50	Sample_42	1	21/05/2018	15/05/2018	King's College	King's College	Clinical	Clinical	31	37	Collection Date of Isolate 2 before Isolate 1
Sample_50	Sample_47	1	21/05/2018	31/05/2018	King's College	King's College	Clinical	Clinical	31	43	
PHE_99	Sample_21	0.98120376	14/10/2016	14/10/2016	King's College	King's College	Clinical	Clinical	17	8	
PHE_23	PHE_101	0.93881224	08/07/2016	25/10/2016	Royal Brompton	King's College	Clinical	Clinical	Unknown	16	
CA_IC24	CA_IC5	0.93661268	08/05/2019	19/04/2019	Centre A	Centre D	Clinical	Clinical	3	2	Collection Date of Isolate 2 before Isolate 1
CA_IC24	CA_IC9	0.9330134	08/05/2019	17/11/2017	Centre A	Centre A	Clinical	Clinical	3	3	Collection Date of Isolate 2 before Isolate 1
CA_IC6	CA_IC4	0.93181364	17/05/2019	03/01/2017	Centre D	Centre D	Clinical	Clinical	2	2	Collection Date of Isolate 2 before Isolate 1
PHE_81	PHE_19	0.90541892	03/10/2016	24/06/2016	King's College	Centre E	Clinical	Clinical	7	Unknown	Collection Date of Isolate 2 before Isolate 1
PHE_81	16I33	0.90541892	03/10/2016	16/06/2016	King's College	Royal Brompton	Clinical	Unknown	7	Unknown	Collection Date of Isolate 2 before Isolate 1
PHE_81	PHE_98	0.90541892	03/10/2016	24/10/2016	King's College	King's College	Clinical	Clinical	7	15	

PHE_81	PHE_12	0.90241952	03/10/2016	11/01/2016	King’s College	Centre E	Clinical	Unknown	7	Unknown	Collection Date of Isolate 2 before Isolate 1
Sample_2	CA_IC10	0.89982004	15/10/2016	05/12/2017	King’s College	Centre A	Clinical	Clinical	7	3	
16B31	PHE_24	0.889822	16/10/2016	08/07/2016	Royal Brompton	Royal Brompton	Unknown	Clinical	Unknown	Unknown	Collection Date of Isolate 2 before Isolate 1
Sample_8	Sample_22	0.8822236	01/11/2016	22/06/2018	King’s College	King’s College	Clinical	Clinical	5	50	
PHE_28	PHE_25	0.86382723	09/03/2016	08/07/2016	Royal Brompton	Royal Brompton	Clinical	Clinical	Unknown	Unknown	
16B4119	16B10136	0.8634273	01/02/2016	15/03/2016	Royal Brompton	Royal Brompton	Clinical	Clinical	Unknown	Unknown	
16B4119	16I5822	0.8634273	01/02/2016	08/02/2016	Royal Brompton	Royal Brompton	Clinical	Clinical	Unknown	Unknown	
Sample_37	Sample_55	0.8620276	18/06/2018	03/07/2018	King’s College	King’s College	Clinical	Environment	49	N/A	
PHE_81	16B9044	0.85342931	03/10/2016	07/03/2016	King’s College	Royal Brompton	Clinical	Clinical	7	Patient C	Collection Date of Isolate 2 before Isolate 1
PHE_64	PHE_66	0.8362328	29/08/2016	08/09/2016	King’s College	King’s College	Clinical	Clinical	4	5	
PHE_64	PHE_55	0.83623275	29/08/2016	22/08/2016	King’s College	Centre E	Clinical	Clinical	4	Unknown	Collection Date of Isolate 2 before Isolate 1
PHE_35	PHE_33	0.835233	28/07/2016	28/07/2016	Royal Brompton	King’s College	Clinical	Clinical	Unknown	3	
CA_IC13	CA_IC11	0.83263347	14/12/2018	14/12/2018	Centre B	Centre B	Clinical	Clinical	4	4	Same collection date, same patient
Sample_12	Sample_4	0.82483503	21/09/2017	06/10/2016	King’s College	King’s College	Clinical	Clinical	34	7	

Sample_10	PHE_82	0.7988402	09/01/2017	05/10/2016	King's College	Centre E	Clinical	Clinical	25	Unknown	Collection Date of Isolate 2 before Isolate 1
Sample_10	Sample_5	0.7988402	09/01/2017	22/06/2017	King's College	King's College	Clinical	Clinical	25	7	
PHE_91	PHE_87	0.790042	30/10/2016	16/10/2016	King's College	King's College	Clinical	Clinical	8	10	Collection Date of Isolate 2 before Isolate 1
PHE_81	PHE_35	0.77864427	03/10/2016	28/07/2016	King's College	Royal Brompton	Clinical	Clinical	7	Unknown	Collection Date of Isolate 2 before Isolate 1
CA_IC2	CA_IC6	0.77704459	03/12/2016	17/05/2019	Centre D	Centre D	Clinical	Clinical	2	2	Same patient
CA_IC13	CA_IC18	0.7554489	14/12/2018	01/02/2019	Centre B	Centre B	Clinical	Clinical	4	5	

Supplementary Table 4: Transmission between Clade III isolates using *TransPhylo* with a probability threshold above 75%. The transmission pathway is from isolate 1 to isolate 2 including probability of transmission from isolates, date of isolate collection, hospital, source and patient numbers. Patient numbers correspond to what was provided by each hospital centre.

Transmission -->											
Isolate 1	Isolate 2	Probability of Transmission from Isolate 1 to Isolate 2	Date of Collection Isolate 1	Date of Collection Isolate 2	Hospital Isolate 1	Hospital Isolate 2	Source Isolate 1	Source Isolate 2	Patient Number Isolate 1	Patient Number Isolate 2	Notes
PHE_104	PHE_86	0.8494301	25/10/2016	13/07/2016	John Radcliffe	John Radcliffe	Clinical	Clinical	Unknown	Unknown	Collection Date of Isolate 2 before Isolate 1
PHE_104	PHE_95	0.8494301	25/10/2016	16/10/2016	John Radcliffe	John Radcliffe	Clinical	Clinical	Unknown	Unknown	Collection Date of Isolate 2 before Isolate 1
PHE_104	SRR7976584	0.8494301	25/10/2016	28/11/2016	John Radcliffe	John Radcliffe	Clinical	Clinical	Unknown	Patient_008	
PHE_6	SRR7976616	0.95001	10/06/2014	13/07/2015	Unknown	John Radcliffe	Clinical	Clinical	Unknown	Patient_003	
PHE_85	SRR7976582	0.984803	29/10/2015	24/11/2015	John Radcliffe	John Radcliffe	Clinical	Clinical	Unknown	Patient_004	
Sample_16	Sample_24	0.8956209	18/07/2018	21/07/2018	King's College	King's College	Clinical	Clinical	52	54	
Sample_16	Sample_57	0.8956209	18/07/2018	04/07/2018	King's College	King's College	Clinical	Clinical	52	21	Collection Date of Isolate 2 before Isolate 1
Sample_16	SRR7976548	0.8956209	18/07/2018	30/08/2017	King's College	John Radcliffe	Clinical	Clinical	52	Patient_021	
Sample_16	SRR7976571	0.8956209	18/07/2018	16/05/2017	King's College	John Radcliffe	Clinical	Environmental	52	N/A	Collection Date of Isolate 2 before Isolate 1
SRR7976544	SRR7976563	0.9998	03/04/2017	16/01/2017	John Radcliffe	John Radcliffe	Clinical	Clinical	Patient_024	Patient_014	Collection Date of Isolate 2 before Isolate 1
SRR7976546	SRR7976552	0.77964407	13/02/2017	17/02/2017	John Radcliffe	John Radcliffe	Clinical	Clinical	Patient_006	Patient_019	

SRR7976546	SRR7976570	0.7796441	13/02/2017	29/01/2017	John Radcliffe	John Radcliffe	Clinical	Clinical	Patient_006	Patient_009	Collection Date of Isolate 2 before Isolate 1
SRR7976554	SRR7976551	1	10/02/2017	20/02/2017	John Radcliffe	John Radcliffe	Clinical	Clinical	Patient_018	Patient_020	
SRR7976554	SRR7976564	0.9876025	10/02/2017	10/01/2017	John Radcliffe	John Radcliffe	Clinical	Clinical	Patient_018	Patient_013	Collection Date of Isolate 2 before Isolate 1
SRR7976554	SRR7976596	1	10/02/2017	17/05/2017	John Radcliffe	John Radcliffe	Clinical	Clinical	Patient_018	Patient_028	
SRR7976557	PHE_32	1	25/10/2016	22/04/2016	John Radcliffe	John Radcliffe	Clinical	Clinical	Patient_006	Unknown	Collection Date of Isolate 2 before Isolate 1
SRR7976557	SRR7976542	1	25/10/2016	17/11/2016	John Radcliffe	John Radcliffe	Clinical	Clinical	Patient_006	Patient_007	
SRR7976557	SRR7976573	1	25/10/2016	15/01/2017	John Radcliffe	John Radcliffe	Clinical	Clinical	Patient_006	Patient_006	
SRR7976558	SRR7976572	0.8116377	07/04/2017	10/03/2017	John Radcliffe	John Radcliffe	Clinical	Clinical	Patient_029	Patient_022	Collection Date of Isolate 2 before Isolate 1
SRR7976562	SRR7976575	0.8120376	14/04/2017	05/04/2017	John Radcliffe	John Radcliffe	Clinical	Clinical	Patient_025	Patient_025	Collection Date of Isolate 2 before Isolate 1
SRR7976562	SRR7976583	0.8070386	14/04/2017	04/04/2017	John Radcliffe	John Radcliffe	Clinical	Environmental	Patient_025	N/A	Collection Date of Isolate 2 before Isolate 1
SRR7976562	SRR7976612	0.8120376	14/04/2017	13/02/2017	John Radcliffe	John Radcliffe	Clinical	Clinical	Patient_025	Patient_006	Collection Date of Isolate 2 before Isolate 1
SRR7976565	SRR7976540	0.8546291	27/03/2017	10/02/2017	John Radcliffe	John Radcliffe	Clinical	Clinical	Patient_025	Patient_017	Collection Date of Isolate 2 before Isolate 1
SRR7976565	SRR7976553	0.8546291	27/03/2017	18/03/2017	John Radcliffe	John Radcliffe	Clinical	Clinical	Patient_025	Patient_023	Collection Date of Isolate 2 before Isolate 1

SRR7976565	SRR7976554	0.8546291	27/03/2017	10/02/2017	John Radcliffe	John Radcliffe	Clinical	Clinical	Patient_025	Patient_018	Collection Date of Isolate 2 before Isolate 1
SRR7976565	SRR7976558	0.8116377	27/03/2017	07/04/2017	John Radcliffe	John Radcliffe	Clinical	Clinical	Patient_025	Patient_029	
SRR7976565	SRR7976568	0.8478304	27/03/2017	30/01/2017	John Radcliffe	John Radcliffe	Clinical	Clinical	Patient_025	Patient_010	Collection Date of Isolate 2 before Isolate 1
SRR7976567	SRR7976541	0.96780644	31/05/2017	05/05/2017	John Radcliffe	John Radcliffe	Clinical	Clinical	Patient_022	Patient_033	Collection Date of Isolate 2 before Isolate 1
SRR7976567	SRR7976546	0.7796441	31/05/2017	13/02/2017	John Radcliffe	John Radcliffe	Clinical	Clinical	Patient_022	Patient_006	Collection Date of Isolate 2 before Isolate 1
SRR7976567	SRR7976560	0.96780644	31/05/2017	16/05/2017	John Radcliffe	John Radcliffe	Clinical	Environmental	Patient_022	N/A	Collection Date of Isolate 2 before Isolate 1
SRR7976567	SRR7976561	0.96780644	31/05/2017	10/05/2017	John Radcliffe	John Radcliffe	Clinical	Clinical	Patient_022	Patient_033	Collection Date of Isolate 2 before Isolate 1
SRR7976567	SRR7976603	0.96780644	31/05/2017	24/04/2017	John Radcliffe	John Radcliffe	Clinical	Clinical	Patient_022	Patient_037	Collection Date of Isolate 2 before Isolate 1
SRR7976567	SRR7976609	0.94921016	31/05/2017	29/01/2017	John Radcliffe	John Radcliffe	Clinical	Clinical	Patient_022	Patient_009	
SRR7976576	SRR7976610	1	07/05/2017	10/04/2017	John Radcliffe	John Radcliffe	Clinical	Clinical	Patient_027	Patient_029	
SRR7976576	SRR7976615	1	07/05/2017	31/03/2017	John Radcliffe	John Radcliffe	Clinical	Clinical	Patient_027	Patient_027	Collection Date of Isolate 2 before Isolate 1
SRR7976588	SRR7976590	0.75144971	08/03/2017	15/03/2017	John Radcliffe	John Radcliffe	Clinical	Clinical	Patient_019	Patient_021	
SRR7976593	SRR7976589	1	04/04/2017	17/02/2017	John Radcliffe	John Radcliffe	Environmental	Clinical	N/A	Patient_018	Collection Date of Isolate 2 before Isolate 1

SRR7976593	SRR7976597	1	04/04/2017	05/12/2016	John Radcliffe	John Radcliffe	Environmental	Clinical	N/A	Patient_009	Collection Date of Isolate 2 before Isolate 1
SRR7976608	PHE_104	0.8494301	13/02/2017	25/10/2016	John Radcliffe	John Radcliffe	Clinical	Clinical	Patient_010	Unknown	Collection Date of Isolate 2 before Isolate 1
SRR7976608	PHE_111	0.9940012	13/02/2017	02/11/2016	John Radcliffe	John Radcliffe	Clinical	Clinical	Patient_010	Unknown	Collection Date of Isolate 2 before Isolate 1
SRR7976608	PHE_8	0.886228	13/02/2017	02/02/2015	John Radcliffe	John Radcliffe	Clinical	Clinical	Patient_010	Unknown	Collection Date of Isolate 2 before Isolate 1
SRR7976608	PHE_84	1	13/02/2017	16/10/2016	John Radcliffe	John Radcliffe	Clinical	Clinical	Patient_010	Unknown	Collection Date of Isolate 2 before Isolate 1
SRR7976608	SRR7976604	1	13/02/2017	09/01/2017	John Radcliffe	John Radcliffe	Clinical	Clinical	Patient_010	Patient_012	Collection Date of Isolate 2 before Isolate 1
SRR7976608	SRR7976614	0.9566087	13/02/2017	02/01/2016	John Radcliffe	John Radcliffe	Clinical	Clinical	Patient_010	Patient_005	Collection Date of Isolate 2 before Isolate 1
SRR7976613	SRR7976569	1	25/01/2017	15/02/2017	John Radcliffe	John Radcliffe	Clinical	Clinical	Patient_011	Patient_011	

Supplementary Table 5: Sequential *C. auris* isolates (all Clade I) taken from patients within four London hospitals to investigate microevolution. MIC data can be found in Supp. Table 1.

Hospital Centre	Patient	Isolate	Collection date	Sample site
Centre C	1	CA_IC1	26/07/2016	Groin swab
Centre D	2	CA_IC2	03/12/2016	Urine
		CA_IC3	28/12/2016	Nose swab
		CA_IC4	03/01/2017	Nose swab
		CA_IC5	19/04/2019	Urine
		CA_IC6	17/05/2019	Urine
Centre A	3	CA_IC7	26/10/2017	Exit site swab
		CA_IC8	26/10/2017	Throat swab
		CA_IC9	17/11/2017	Swab no site
		CA_IC10	05/12/2017	Exit site swab
		CA_IC24	08/05/2019	Exit site swab
Centre B	4	CA_IC11	14/12/2018	Axilla swab
		CA_IC12	09/12/2018	Groin swab
		CA_IC13	14/12/2018	Groin swab
	5	CA_IC14	22/11/2018	Sputum
		CA_IC15	30/12/2018	Nose swab
		CA_IC16	30/12/2018	Groin swab
		CA_IC17	04/01/2019	Bronchial wash
		CA_IC18	01/02/2019	Urine
		CA_IC20	04/02/2019	Urine
	6	CA_IC19	05/02/2019	Rectal swab
		CA_IC21	05/02/2019	Groin swab
		CA_IC22	19/02/2019	Groin swab
		CA_IC23	19/02/2019	Urine
